# The Effectiveness of Affirmative Sexual Consent Cues

**DOI:** 10.1177/08862605251343200

**Published:** 2025-06-03

**Authors:** Krusha Upadhyay, Tiffany Lavis, Michael Proeve

**Affiliations:** 1 The University of Adelaide, SA, Australia

**Keywords:** sexual consent, victim blame, sexual script theory, implicit nonverbal cues, explicit nonverbal cues

## Abstract

Despite the growing literature related to sexual consent, most research has focused on female victims, indicating a significant need for more research focusing on consent and victim blame with male victims. The current study investigated whether male victims are blamed for sexual assault where the perpetrator is a female, using traditional sexual script theory as a framework. The study investigated whether participants could clearly differentiate between consent and non-consent scenarios when ambiguous nonverbal cues were presented. Participants (*N* = 167) were randomly assigned to one of three vignettes in a between-subjects experimental design: (a) A neutral control condition with no clear cues, (b) implicit nonverbal non-consent cues, and (c) explicit nonverbal non-consent cues. Participants were asked to rate consent and victim blame. Overall, the findings indicated that male participants placed more blame on victims and rated all scenarios (regardless of implicit or explicit cues) as more consensual than female participants. Furthermore, the findings of this study suggest that participants can identify non-consent, if any sort of cue (implicit or explicit) is presented. Nonetheless, participants believe that explicit cues, rather than implicit cues, would be the most effective method of communicating non-consent. These findings suggest that education campaigns may benefit from incorporating ambiguous non-consent cues in discussions about how consent is communicated.

## Introduction

The field of research which considers male sexual assault victims is under-researched, with some researchers estimating it to be approximately 20 years behind research on female sexual assault victims (Abbey, 2002; Mauck, 2016; Sleath & Bull, 2010). However, we know that traditional sexual scripts perpetuate gender stereotypes, and therefore, men and women may have different expectations regarding how consent or non-consent cues should be communicated or interpreted (Jozkowski et al., 2014; Parker, 2023). According to sexual script theory, individuals create meaning from sexual behaviors that are influenced by the shared beliefs of others. In a conceptual review of the literature, gender was identified as the most crucial component related to sexual consent communication, particularly affirmative consent (Willis et al., 2019). Men are more likely to take on the role of requesting consent, whereas women are expected to decide whether to engage in sexual activity (Cowling & Reynolds, 2016). The behavioral and interpretation differences are shaped by the attitudes and beliefs (sexual script) that each individual conveys in the sexual encounter.

In traditional sexual scripts, sexual interest by both men and women is typically communicated in subtle, implicit ways, rather than through direct verbal consent (Jozkowski et al., 2014). For example, women’s sexual scripts have socialized them to be passive and communicate indirectly (Newstrom et al., 2020; Wiederman, 2005). Misinterpretation can occur when one individual interprets cues as consent, while the other does not believe they are communicating consent. Previous research suggests that male participants may perceive (women’s) nonverbal cues inaccurately (Henningsen et al., 2006; Jozkowski et al., 2014; Marcantonio & Jozkowski, 2021; Newstrom et al., 2021). For instance, even when a woman may not be consenting to engage in sexual activity, a man may perceive nonverbal cues as consent; and when this sort of miscommunication occurs between genders, it may result in an unwanted or coercive sexual encounter (Henningsen et al., 2006; Jozkowski et al., 2014). These findings should not be interpreted to imply that men are always willing perpetrators in such situations, though this may be the case for some; rather, it points to the possibility of non-consent miscommunications between men and women (Jozkowski et al., 2014).

As sexual consent requires the communication of sexual intention, which can be communicated both verbally and nonverbally, certain cues may be more likely to be misinterpreted than others (Winslett & Gross, 2008). Hickman and Muehlenhard (1999) discovered five patterns of sexual consent cues using factor analysis: direct verbal cues (e.g., “will you have sex with me?”), direct nonverbal cues (e.g., taking clothes off), indirect verbal cues (e.g., asking to get a condom), indirect nonverbal cues (e.g., touching sexually), and statements about intoxication levels (e.g., says “I am really drunk”) (p. 264). In a previous study, non-consent was defined using explicit verbal behaviors; for instance, “verbally saying no,” or “nonconsent is when the partner doesn’t say no but also never says yes” (Marcantonio & Jozkowski, 2021, p. 9). Establishing sexual consent is a complex process, where indicators of consent are evaluated throughout the sexual encounter, and indicators are potentially impacted by a number of situational factors (see, e.g., Edwards et al., 2024; Pazos et al., 2024). However, there have been some suggestions that discussion of sexual consent may “ruin the mood”, be gender incongruent, or fall outside of individuals’ normative sexual scripts (Humphreys, 2007; Humphreys & Herold, 2007). As a result, individuals may rely on indirect behaviors to indicate a willingness to engage in sexual interaction (Gangi, 2017; Hardesty et al., 2022; Righi et al., 2021).

Many qualitative studies show that implicit nonverbal acts are used to demonstrate consent (Beres, 2010; Bindesbøl Holm Johansen et al., 2020; Hien, 2008; Ólafsdottir & Kjaran, 2019; Righi et al., 2021). These cues are usually the same regardless of whether men or women initiate sexual activity (McCormick, 2010). However, miscommunication occurs when these nonverbal cues are perceived differently between men and women. Nonverbal cues that can be interpreted as both sexual interest and politeness (e.g., smiling and eye contact) can be difficult to differentiate in opposite-sex interactions (Gangi, 2017). Several studies show that men, on average, attribute more sexual meaning to women’s behavior than women intend (e.g., Henningsen et al., 2009; Levesque et al., 2006; Parker, 2023). For instance, when Vrij and Kirby (2002) asked college students to view a muted videotape of a man and a woman conversing, men attributed more sexual intent to the interactants than women. These studies suggest that the interpretation of consent or non-consent may be imprecise, with men incorrectly assuming that consent has been granted (Gangi, 2017).

Previous research has begun to explore how sexual cues may be observed in establishing affirmative consent. For example, Mattson et al. (2022) asked college students to rate behaviors (e.g., actively touching partner; avoiding eye contact), to determine whether they were indicative of affirmative sexual consent. The behaviors were collated through a review of the literature and discussions with students. The overarching aim of the study was to categorize students into one of three groups, depending on how they viewed affirmative consent: restrictive, inclusive, nondiagnostic. Findings indicated that students were either restrictive or inclusive, related to past sexual experiences, attitudes, and education (Mattson et al., 2022). This research indicated that there may be differences in how the affirmative consent policy is interpreted.

Similarly, Harrell et al. (2023) explored what college students believe constitutes nonverbal affirmative sexual consent. This qualitative study interviewed cisgender participants, who self-selected to participate, about nonverbal indicators of sexual consent. Participants were asked to write two fictional scenarios that depicted how consent might be obtained through nonverbal communication, and then these scenarios were explored through discussion in focus groups. There was a set of common behaviors that were identified. The authors categorized these in terms of “invasiveness”, ranging from “nodding” (minimal invasiveness), to “exposing one’s genitals” (moderate to significant), to “removing another individual’s clothing” (significant) (Harrell et al., 2023, p. 242). There was consistency noted between the behaviors identified and previous literature (see, e.g., Hickman & Muehlenhard, 1999; Jozkowski et al., 2014). However, the research acknowledged that contextual factors, generated by individual participants, made it difficult to assess nonverbal affirmative cues. This highlights the need for a quantitative approach to this area of research.

More recently, Parker (2023) used a quantitative approach to explore how college students navigate sexual consent. This study considered how individual behaviors are used to indicate sexual interest. The behaviors were chosen on the basis of the authors’ review of the literature and were categorized to range from interest to disinterest. Participants were asked to rate their sexual interest and desire on the basis of the behaviors. Overt behaviors (i.e., saying yes) were, not surprisingly, found to indicate sexual interest. Consistent with previous literature, men were found to rank several behaviors as more indicative of sexual interest when compared with women (e.g., prior to sex, pinned partner down; prior to sex, kissed partner’s neck). A second study by Parker (2023) explored contextual factors (e.g., relationship type, behaviors prior to and during sexual encounter, the number of behaviors demonstrated). Participants were presented with four vignettes depicting heterosexual sexual encounters. Information which might indicate sexual disinterest (e.g., remaining still; saying no) was, not surprisingly, found to impact perceptions of sexual disinterest in terms of sexual consent. This research further indicates the complexity of sexual consent and the importance of determining how the individual cues are perceived among the “noise” of other cues that form part of sexual encounters.

The primary purpose of the current study was to examine how individuals respond to implicit and explicit nonverbal cues; specifically, to explore whether participants would identify ambiguous nonconsensual cues in the context of understanding sexual assault as a potential miscommunication because of nonverbal communication. A further objective was to determine which non-consent cues would be rated as “effective” in communicating non-consent. Additionally, this study sought to consider the role of gender, given that previous research findings indicate men may rate behaviors as more indicative of consent than women (Hickman & Muehlenhard, 1999; Humphreys, 2007; Parker, 2023), including interpreting consent from implicit nonverbal acts (Jozkowski et al., 2014, 2018; Newstrom et al., 2021; Willis & Jozkowski, 2022). We also sought to consider how gender may bias perceptions of sexual assault victims, given that previous research has indicated heterosexual men may blame male sexual assault victims when the perpetrators are female (Davies et al., 2006). Thus, through the lens of sexual script theory, we considered how participants perceive sexual assault when the traditional scenario of a female consenting (or non-consenting) is flipped to a male consenting (or not consenting).

## Methods

### Participants

Participants were recruited from a leading research-intensive Australian university in a city of approximately 1.4 million people. There were 148 participants (108 women, 36 men, 3 nonbinary, 1 transgender female) included in the study as either volunteers or in exchange for course credit. The recruitment procedure included advertising the study (“Who is to blame? Perceptions of affirmative sexual consent”) through a university website and participation system, which enabled students to self-select to participate in this study. The final sample included participants aged between 18 and 30, with an average age of 19.78 years (*SD* = 2.66). The sample was predominantly identified as Caucasian (66.2%), followed by Asian (23.6%), and African (1.4 %), with the remaining participants identifying as “other” (8.8%). Most participants reported being single (54.7%), with the remaining participants indicating they were in a relationship (41.2%) or living with a partner (4.1%). The majority of participants reported their sexual orientation as heterosexual (65.5%), followed by bisexual (21.6%), homosexual (4.7%), other (4.7%), and “prefer not to say” (3.4%). Participants were randomly assigned to one of three levels of the nonverbal refusal cues (consent, implicit, explicit).

### Design and Procedure

Ethical approval was obtained from the University of Adelaide Human Research Ethics Committee prior to data collection. Most previous research has used primarily qualitative interviews to investigate how participants would indicate affirmative sexual consent and refusal. However, the present study aims to compare differences in types of cues for effective communication of consent, as well as differences in perception of cues based on gender. Therefore, a quantitative approach was considered appropriate. Specifically, the study focused on nonverbal non-consent cues, as previous research has demonstrated that verbal non-consent cues are more clearly identified. The study aimed to test the degree to which participants identified nonverbal cues of non-consent as indicating non-consent. In addition, this study intended to investigate the degree to which a male is blamed for sexual assault, because of traditional sexual script theory, by reversing the traditional scenario from a female victim to a male victim.

The vignettes were designed for this study to produce the manipulations necessary to test the various interactions. Specifically, the vignettes included refusal cues outlined by Hickman and Muehlenhard (1999). The refusal cues were limited to nonverbal cues, consistent with the study’s aims. In line with the categorizations outlined by Hickman and Muehlenhard (1999), the current study utilized both explicit (“direct”) and implicit (“indirect”) refusal cues (p. 264). For example, explicit nonverbal cues included *taking one’s clothes off*, whereas implicit nonverbal cues included *touching the other individual sexually* (Hickman & Muehlenhard, 1999). One vignette presented a sexual interaction between two individuals (Amy and Jeff), with an absence of clear cues to indicate either consent or non-consent. A second vignette included explicit nonambiguous nonverbal refusal cues demonstrated by the male individual (Jeff), including Jeff physically moving Amy’s hand away, physically pushing Amy away, and shaking his head “no”. The third vignette included implicit, ambiguous nonverbal refusal cues demonstrated by the male individual (Jeff), including Jeff attempting to keep his clothes on, avoiding eye contact, and making a facial expression of disinterest. Participants each read a vignette which presented one of the three experimental vignettes (control, explicit, implicit), involving a sexual interaction with a male (victim) and female (perpetrator). Participants were asked to respond to a series of questions after the vignette.

Participants were asked to make a judgment about whether they perceived the sexual interaction to be consensual. To gain an insight into the specific refusal cues participants thought were effective, indicating non-consent, a single question asked participants to rank the effectiveness of refusal from *most effective* (6) to *least effective* (1). Finally, perceptions of consent were calculated by averaging 13 items measuring the extent to which each individual consented to the sexual interaction (e.g., “To what extent did Jeff provide consent for sexual intercourse?”). The resultant consent measure was found to have good internal consistency (α = .79). We also explored whether participants accurately attended to the vignettes.

## Results

### Manipulation Check

Participants generally identified information from the vignettes (i.e., who initiated the kiss), which indicated they had correctly attended to what had occurred. When no refusal cues were demonstrated in the vignette, 100% of participants correctly identified the information. However, 14% of participants incorrectly identified the information where explicit refusal cues were included in the vignettes, and 6% of participants incorrectly identified the information where implicit refusal cues were included in the vignettes.

### Dependent Variables

Three main issues were examined in this study. First, perceptions of sexual consent were explored. Descriptive statistics for consent are shown in Table 1. The analyses sought to test whether the absence of refusal cues would result in greater perceptions of consent (when compared with implicit or explicit refusal cues), and whether indirect and subtle cues (i.e., implicit cues) would be perceived as consent more than clear, unambiguous cues (i.e., explicit cues). Further, the analyses sought to test whether there were any differences in perceptions of sexual consent based on gender.

**Table 1.** Means (and Standard Deviations) for Perceived Sexual Consent for Participant Gender Across Refusal Cues Conditions.

Measure	Refusal Cues		
	Control	Implicit	Explicit
Female	32.94 (5.46)	25.97 (4.59)	25.89 (5.55)
Male	39.60 (5.73)	30.80 (7.76)	28.58 (6.71)
Overall	35.02 (6.31)	26.52 (5.16)	26.57 (5.91)

A 2 (participant gender: female, male) × 3 (refusal cues: control, implicit, explicit) ANOVA was conducted to explore perceived sexual consent. There was a significant main effect of refusal cues, *F*(2, 133) = 29.27, *p* < .001, η^2^ = .31. Post hoc comparisons using the Scheffe test indicated that the sexual encounter with no clear cues (control condition) was perceived as more consensual than the sexual encounters where either implicit (*p* < .001, 95% CI [5.67, 11.33]) or explicit (*p* < .001, [5.66, 11.23]) refusal cues were demonstrated. There was no significant difference for perceived sexual consent between the sexual encounter with implicit refusal cues and the sexual encounter with explicit refusal cues (*p* = .999, [−2.79, 2.90]). Additionally, there was a significant main effect for gender, *F*(1, 133) = 15.40, *p* < .001, η^2^ = .10, indicating that male participants rated the sexual interactions as more consensual than female participants. The interaction between gender and refusal cues was nonsignificant, *F*(2, 134) = 0.03, *p* = .973, η^2^ = .018.

Second, perceptions of victim blame were explored. Table 2 shows the descriptive statistics for the analyses of victim blame according to gender and nonverbal refusal cues. A 2 (participant gender: female, male) × 3 (refusal cues: control, implicit, explicit) ANOVA was conducted to explore perceptions of victim blame. As anticipated, there was a significant main effect of refusal cues, *F*(2, 136) = 12.01, *p* < .001, η^2^ = .15. Post hoc comparisons using the Scheffe test indicated that the victim blame was significantly higher where the sexual encounter included no clear refusal cues, when compared with the sexual encounter which included implicit refusal cues (*p* = .008, 95% CI [1.04, 8.29]), and also when compared with the sexual encounter which included explicit refusal cues (*p* < .001, [4.82, 12.03]). Additionally, there was a statistically significant difference between the sexual encounters which included explicit and implicit refusal cues, with participants rating victim blame significantly higher where implicit refusal cues were included in the sexual encounter when compared with explicit refusal cues (*p* = .043, [−7.43, −.10).

**Table 2.** Means (and Standard Deviations) for Victim Blame for Participant Gender Across Refusal Cues Conditions.

Measure	Refusal Cues		
	Control	Implicit	Explicit
Female	29.41 (7.05)	24.59 (7.29)	20.80 (7.67)
Male	32.87 (5.76)	32.57 (7.14)	25.67 (6.76)
Overall	30.47 (6.81)	25.80 (7.75)	22.04 (7.68)

**Figure 1.**
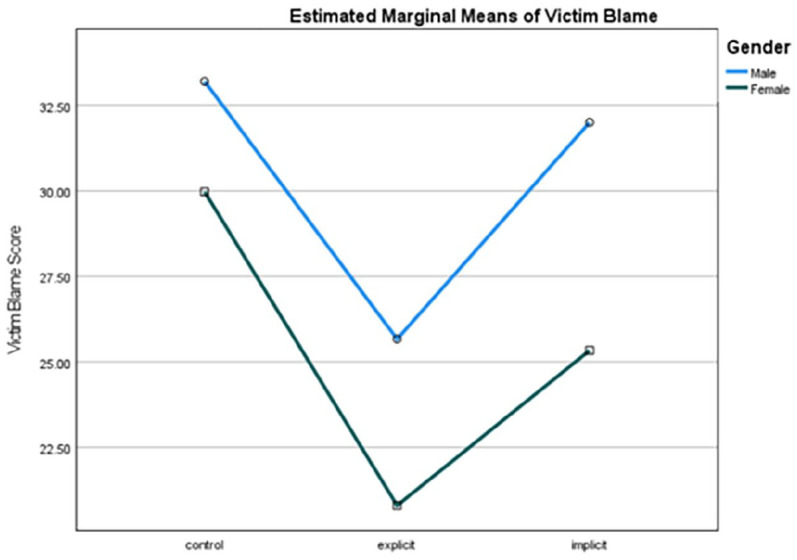
Victim blame scores across participant gender and refusal cues.

There was also a significant main effect for participant gender, *F*(1, 136) = 13.86, *p* < .001, η^2^ = .09. This indicated that perceived victim blame ratings were significantly higher when made by male participants when compared with the ratings made by female participants (see Figure 1). The interaction between gender and cue condition was not statistically significant, *F*(2, 136) = 0.76, *p* = .468, η^2^ = .011.

Finally, the effectiveness of sexual cues in communicating affirmative non-consent was explored. To test the effectiveness, means were calculated from the rankings, with a higher number indicating greater effectiveness of a cue in demonstrating non-consent. Table 3 outlines the means and standard deviations for the participants’ rankings of the effectiveness of sexual cues. Notably, participants rated the three explicit cues higher in terms of effectiveness in communicating consent when compared with rankings for the three implicit cues. The (explicit) cue that was rated as most effective in communicating consent was “physically pushing someone away”. The least effective (implicit) cue was “avoiding eye contact”.

**Table 3.** Means (and Standard Deviations) for Participants’ Rankings of Effectiveness of Sexual Cue in Communicating Non-consent.

Refusal Cues	
Explicit cues	
Physically pushing someone away	5.01 (1.75)
Shaking head “no”	4.01 (1.29)
Physically moving another person’s hand	3.90 (1.16)
Implicit cues	
Attempt to keep clothes on	3.44 (1.04)
Facial expression of disinterest	2.60 (1.43)
Avoiding eye contact	2.17 (1.77)

## Discussion

The purpose of this study was to investigate ambiguous non-consensual sexual cues, to determine whether there are appreciable differences between implicit and explicit nonverbal cues, and whether gender impacts the interpretation of these cues. Specifically, through the lens of sexual script theory, this study aimed to explore how participants perceive non-consent when males are victims of sexual assault. As such, this is one of the very few studies that has explored perceptions of victim blame in heterosexual relationships where the traditional sexual script has been reversed, with the male victimized. As anticipated, male participants placed more blame on the male victims when compared with female participants. This finding is consistent with previous research, which suggests that male participants attribute more blame to male victims when compared with females (Davies & Rogers, 2006). Additionally, male participants rated the sexual interactions as more consensual when compared with female participants. This is also consistent with previous research and the viewpoint that male participants may perceive the nonverbal cues inaccurately (Henningsen et al., 2006; Jozkowski et al., 2014). However, the current study adds to the previous research, indicating that regardless of the cue type presented in the hypothetical vignette (control, implicit, or explicit), male participants rated the sexual encounter as more consensual (see, e.g., Parker, 2023).

As anticipated, perceptions of both consent and victim blame were impacted by the presence or absence of non-consensual sexual cues. Specifically, where there were no clear cues (in the control condition), the interaction was perceived as more consensual and victim blame was significantly higher, when compared with the sexual encounters which included either implicit or explicit non-consensual cues. The present study extends previous research (see, e.g., Beres, 2010; Bindesbøl Holm Johansen et al., 2020; Hien, 2008; Ólafsdottir & Kjaran, 2019; Righi et al., 2021) through quantitative results that male and female participants did not perceive implicit cues as more consensual, even if they are subtle. Although perceptions of consent were not significantly different between implicit and explicit cues in the current study, the perceptions of victim blame did vary. As anticipated, victims were significantly more likely to be blamed where implicit refusal cues were included in the sexual encounter when compared with explicit refusal cues. This finding is consistent with the ratings of effectiveness of the sexual cues in communicating affirmative non-consent. Explicit cues (e.g., physically pushing someone away) are seen as more effective in communicating consent whereas implicit cues (e.g., avoiding eye contact) are seen as less effective. However, nonverbal signs of non-consent were identifiable. Taken together, these findings indicate that there is an expectation that individuals will demonstrate explicit cues and will otherwise be more likely to be blamed for the resulting sexual interaction. These findings are particularly notable in light of previous research, which indicates more implicit sexual cues have traditionally been more commonly used to demonstrate consent (Beres, 2010; Bindesbøl Holm Johansen et al., 2020; Hien, 2008; Ólafsdottir & Kjaran, 2019; Righi et al., 2021), irrespective of the gender of the individual initiating sexual activity (McCormick, 2010). This highlights the importance of affirmative sexual consent campaigns.

The current findings add to the research that has begun to consider the behaviors that constitute affirmative sexual consent (e.g., Harrell et al., 2023; Mattson et al., 2022; Parker, 2023). The approach taken in the current study differs from that of Mattson et al. (2022), in that behaviors assessed in this study were indicative of expressing consent refusal, rather than affirming consent. As an example, Mattson et al. (2022) asked participants to consider the impact of “smiling”, whereas in the current study, we consider how a “facial expression of disinterest” might be interpreted. The interpretation of these more ambiguous behaviors is potentially what creates risk in sexual encounters. Exploring how individuals can learn to express and interpret non-consent, especially where cues might be more ambiguous, is important for both parties in a sexual encounter.

The current study also expands the findings of Harrell et al. (2023), who explored perceptions of common behaviors used to represent consent. Similar to the approach by Mattson et al. (2022), Harrell et al. (2023) consider behavioral indicators of affirmative consent, whereas our study has explored how non-consent might be expressed. However, there were similarities to the behavioral cues used in this study, examined from the perspective of non-consent. For example, Harrell et al. (2023) consider “nodding” to be a minimally invasive high-strength indicator. In our study, “shaking head ‘no’”, which may be regarded as the converse of nodding, was an explicit nonverbal cue for non-consent. Similarly, “removing another individual’s clothing” was a significantly invasive high-strength indicator (Harrell et al., 2023), which conversely aligns with “attempt to keep clothes on”, which was an implicit nonverbal cue in the current study. Taken together, the research begins to build a picture of how affirmative consent might be given or refused, based on a consistent set of behaviors. One of the main differences between the studies was that Harrell et al. (2023) asked individuals to formulate consent from their own experiences or ideas related to consent, whereas the current research considered responses to scenarios that were presented to the participants. Therefore, our findings might be useful in settings such as criminal justice, where understanding the complexity of interpreting affirmative consent is relevant. However, it is interesting to observe the consistency of the findings between the two studies, which indicate the need for further research concerning additional behaviors that might be interpreted to represent non-consent.

Further, the current research expands the previous study of Parker (2023), which considered behaviors that were indicative of disinterest (and interest), where participants self-rated how the behaviors would impact their level of interest and desire. There was some consistency in the types of behaviors that were considered in both of our studies (e.g., avoided eye contact). However, the behaviors tended to indicate varying levels of consent or ambivalence (e.g., kissed, hugged, verbally agreed to engage in the act), and Parker (2023) did not explicitly consider how these behaviors might indicate non-consent. Our study explored the relative strength of the behaviors that represent non-consent. For example, we quantitatively established how participants are likely to rate cues, in order to begin to understand how an escalation of cues might indicate non-consent. This enabled us to begin to draw conclusions about how cues (i.e., avoiding eye contact) might be interpreted, directly compared with other cues (i.e., pushing someone away).

### Limitations

One major limitation of the current research is that the study sought to establish consent and victim blame on the basis of a brief sexual interaction, where measurements were obtained at a single point in time. Sexual consent is complex, and several qualitative studies have indicated that participants perceive consent communication as a process (Beres, 2010; Humphreys, 2004; Jozkowski et al., 2018). For example, Willis and Jozkowski (2022) suggest that participants may become increasingly more confident as cues are presented. While many of these misunderstandings about sexual interest or consent between males and females are quickly corrected, others are not, and this may result in miscommunication and ultimately sexual coercion. Further, as men have been socialized to initiate sexual intimacy and to dominate women to obtain what they desire (see, e.g., Jozkowski et al., 2014), messages such as these may make men less likely to regard the presence of non-consent as a problem, especially in a scenario with a male victim. The current study does not account for situational factors, including how ambiguous cues are interpreted in the context of factors such as alcohol consumption and relationship type (see, e.g., Pazos et al., 2024). As such, the snapshot approach used in the current study is potentially a limitation, given that it removes much of the contextual information. Future studies could also consider the impact of a single cue and whether there is a difference with a sequence of non-consent cues, especially where there are situational factors. Additionally, recent research has considered factors that differ from the affirmative consent view (see, e.g., Edwards et al., 2024, for discussion about consent as internal desire and lack of coercion), which indicates there is further complexity to establishing sexual consent that requires consideration.

### Conclusion

The current study addresses gaps in previous research by exploring perception of consent and victim blame in a scenario with female perpetrators and nonverbal refusal cues. One of the primary goals of this research was to focus on the under-researched area of sexual assault, specifically with female perpetrators and their effects on gender differences and their views on consent perception and victim blame. Overall, the findings of this study have implications for understanding how participants perceive, rate, and understand non-consensual sexual encounters and specifically gender differences in consent and victim blame. Specifically, male participants were found to rate scenarios (regardless of implicit or explicit) as more consensual than female participants and place more blame on victims. In addition, it was discovered that sexual encounters with neutral conditions (no clear cues) were perceived as more consensual than those with implicit or explicit refusal cues. These findings suggest that if cues (implicit or explicit) are present, individuals can identify non-consent. However, regardless of their ability to identify implicit and explicit non-consent cues, participants rated explicit cues (physical cues) as a stronger predictor of refusal than implicit cues (behavioral cues). The findings of this study can be used in future research to investigate the sexual perception of female perpetrators and to promote educational interventions to encourage understanding of subtle, nonverbal cues to reduce sexual assault. It is suggested that future research investigate a more in-depth sexual encounter vignette in which participants are presented with various cues that could indicate clear non-consent, to see if their perspectives on consent differ. Future research should also consider the complexities of consent and blame, exploring whether there is a minimum threshold for non-consent to be established, whether there are ordering effects, and whether cues may be diluted in the context of extraneous variables.
